# Magnitude of discordance between registry data and death certificate when evaluating leading causes of death in dialysis patients

**DOI:** 10.1186/1471-2288-13-51

**Published:** 2013-03-27

**Authors:** Jean-Philippe Lafrance, Elham Rahme, Sameena Iqbal, Martine Leblanc, Vincent Pichette, Naoual Elftouh, Michel Vallée

**Affiliations:** 1 Centre de recherche Hôpital Maisonneuve-Rosemont, Montréal, Canada; 2 Département de Médecine, Université de Montréal, Montréal, Canada; 3 Service de néphrologie, Hôpital Maisonneuve-Rosemont, 5415, boul. de l’Assomption, Montréal (Québec), H1T 2M4, Canada; 4 Department of Medicine, McGill University, Montreal, Quebec, Canada; 5 Research Institute, McGill University Health Centre, Montreal, Quebec, Canada; 6 Division of Nephrology, McGill University Health Centre, Montréal, Québec, Canada

**Keywords:** Registries, Renal dialysis, Kidney failure, Chronic, Mortality, Cardiovascular diseases, Infections

## Abstract

**Background:**

Discordance between dialysis registry and death certificate reported death has been demonstrated. Since cause of death is measured using registry data in dialysis patients and death certificate data in the general population, comparisons of cause of death proportions between dialysis patients and the general population may be biased. Our aim was to compare the proportion of deaths attributed to cardiovascular disease (CVD), malignancy, and infections between patients receiving dialysis and the general population using death certificates for both, and to quantify the magnitude of discrepancy between registry and death certificate estimates in dialysis patients.

**Methods:**

A retrospective cohort study of 5858 patients initiating maintenance dialysis between 2001 and 2007 was conducted. Cause of death was obtained from both registry and death certificate data for dialysis patients, and from death certificate data for the general population.

**Results:**

Compared to the general population, use of death certificate data in dialysis patients resulted in smaller differences in the proportion of deaths attributed to CVD or infection than that from the registry. In the general population, the proportion of deaths due to CVD is 29.3% for men and 28.2% for women, and the proportion of deaths due to infection is 3.3% for men and 3.6% for women. For men, the proportion of deaths in dialysis patients due to CVD using registry data is 41.5%, compared with a proportion of 32.1% using death certificate data. Similarly for women, the proportion of deaths due to CVD using registry data is 35.2% and that using death certificate data 24.3%. The proportion of deaths due to infection in dialysis patients follows the same pattern: for men, the proportion of deaths due to infection using registry data is 9.9% and that from death certificate data at 5.0%; while for women the proportions are 11.6% and 4.8%, respectively.

**Conclusions:**

While absolute cause-specific mortality rates did differ, evaluation of causes of death using death certificate in dialysis patients in Quebec revealed that they do not have substantially different proportion of death due to CVD or infections than the general population. Infections appeared to be a frequent complication leading to death, suggesting that infections are an important target to consider for reducing mortality in dialysis populations.

## Background

In Canada, more than 22 000 patients were receiving chronic dialysis in 2009, increasing by nearly 20% in five years [1]. Cardiovascular disease (CVD) is the leading cause of mortality among dialysis patients, followed by infections [1]. It is well-established that dialysis patients have a higher mortality rate than the general population (GP) [1-3], and some data suggest that they have a higher proportion of death due to CVD than the GP [4,5].

Understanding causes of death in a given population is important, as this data may guide prevention efforts, patient management, and clinical or research resources allocation. Many countries, including Canada, rely on registry data to produce estimates of causes of death for their dialysis population. In the GP, death certificates are considered the best practical source of truth at a population level. Since cause of death is measured using registry data in dialysis patients and death certificates in the GP, comparisons of cause of death proportions between dialysis patients and the GP may be biased. Reports comparing death certificates with dialysis registry data have demonstrated poor cause of death concordance [6,7]. However, the quantification of the difference relative to GP estimates has not been undertaken. Using a single source of truth, such as death certificates, through linkage of databases may produce more reliable estimates and may drive better clinical, research and administrative decisions.

Interestingly, while many CVD-oriented interventions such as use of statins [8,9] or angiotensin converting enzyme inhibitors [10] have resulted in some improvement in outcomes (such as reduction in atherosclerotic events), none have been demonstrated to lower mortality in the dialysis population. While pathogenic processes for CVD are probably different in the dialysis population compared to the GP, the inability of large studies to demonstrate improved survival, may suggest that attention to CVD deaths alone is insufficient to reduce mortality. Attention to other important contributors, such as infections, may be important [3]. In order to inform decision-making more accurately concerning intervention targets, assessment of causes of death proportions among dialysis patients should be ascertained using the same source of truth as in the GP. Our aim was to compare the proportion of deaths attributed to CVD, malignancy, and infections between patients receiving dialysis and the GP using death certificates for both, and to quantify the magnitude of discrepancy between registry and death certificate estimates in dialysis patients.

## Methods

### Data sources and study population

Data were obtained from the national Canadian dialysis registry, the Canadian Organ Replacement Register (CORR), and the provincial health services administrative databases of the province of Québec, Canada. CORR provides descriptive statistics on dialysis incidence, prevalence and patients’ characteristics, and its data have been used successfully in numerous scientific publications [11-15].

All Québec residents, more than 8 million inhabitants, are covered for their physician and hospital services by a universal single-payer health care system (*Régie de l’assurance maladie du Québec* – RAMQ). The RAMQ physician claim databases include all visits, diagnosis codes and procedures during in- or outpatient encounters. RAMQ also hosts the hospital discharge summary databases. The *Institut de la statistique du Québec* (ISQ) holds official governmental vital statistic databases, which include dates and causes of death as reported on the death certificate. Information on data sources is summarized in Table 1.

**Table 1:** Data sources

	**Canadian organ replacement register (CORR)**	***Institut de la statistique du Québec *(ISQ)**	***Régie de l’assurance maladie du Québec *(RAMQ)**
Type	Dialysis registry	Provincial vital statistics	Medical claims and hospital discharge summaries database
Population included	All chronic dialysis patients in Canada	All residents in Québec (general population)	All residents in Québec (general population)
Filling of cause of death field	Registered nurse responsible for the unit, helped with treating physician	Treating physician	N/A
Cause of death coding scheme	Internal coding scheme (code entered directly on the reporting form)	ICD-10 by trained archivists centrally	N/A

Abbreviations: ICD-10, *International Classification of Diseases, 10th Revision.*

From CORR, RAMQ and ISQ, data were obtained for all patients initiating chronic dialysis (without a prior kidney transplant) between January 1st, 2001 and December 31st, 2007 in the province of Québec. Patients with less than 90 days of dialysis were excluded. The study cohort consisted of all patients who were present in both the CORR and RAMQ databases as incident dialysis patients. An incident cohort was used, since comorbidities and causes of death may highly depend on dialysis vintage. Patients were followed from day 90 after dialysis initiation until date of death or end of the study period.

Mortality rates in the GP of Québec were obtained from the ISQ website for the years 2001 to 2007 [16].

### Measurement of dates and causes of death

CORR data provided a date of death (month and year) and a cause of death using an internal classification (78 elements). The cause of death is usually coded by the registered nurse responsible in each dialysis unit.

ISQ also provided a date of death (month and year) and a cause of death coded using the *International Classification of Diseases, 10th Revision* (ICD-10). Death certificates are filled by physicians and then coded by trained archivists at ISQ. For the evaluation of dates of death concordance, the date of death provided by RAMQ-ISQ was considered the source of truth.

The cause of death is mandatory on the death certificate (ISQ) and includes different fields: 1) underlying disease that eventually led to death; 2) diseases in the pathway to death (“secondary causes”); and 3) the disease or complication that directly led to death (“direct cause”). For example, a patient may have the following pathway: had an acute myocardial infarction (underlying cause), followed by a cardiogenic shock (secondary cause), and dies after a ventilator-associated pneumonia in the intensive care unit (direct cause).

Causes of death were classified in four mutually exclusive categories: CVD (ICD-10: I00-I99), infection (A00-B99, J10-J18), malignancy (C00-D48), and other. Among the “other” category, kidney failure (N17-N19) and diabetes (E10-E14) were identified using death certificate, but those categories had no code using CORR internal scheme.

### Statistical analysis

Dates of death from CORR and ISQ were considered concordant if they occurred in the same month, or in a contiguous month. Concordance was measured using kappa statistics.

Mortality rates were calculated by dividing the number of deaths by the total patient-years of follow-up. 95% confidence intervals (CI) for rates were calculated using a Poisson distribution. Mortality rates for the GP were indirectly standardized using the study cohort age and sex structure. Cumulative survival function was calculated using Kaplan-Meier method.

Causes of death were considered concordant if they fell within the same category. Two concordance analyses were done for causes of death: 1) CORR *versus* ISQ underlying cause and 2) CORR *versus* ISQ direct cause. Concordance was measured by non-weighted kappa statistics for categorical variables [17], and by a Chi-square test for proportions.

### Sensitivity analyses

Some codes in CORR classification system are broad and may include various categories: *Cardiac Arrest, Cause Unknown*; *Patient Refused Further Treatment*; *Multi System Failure*; or *Other Identified Cause of Death*. In the main classification, theses codes were classified as “Other” except *Cardiac Arrest, Cause Unknown* that was classified as CVD. To test the impact of this decision on the results, a sensitivity analysis was conducted by excluding patients who had one of these codes. Also, because a large proportion of causes of death were missing in CORR, two sensitivity analyses were conducted where all missing causes were attributed to 1) CVD or 2) other causes.

### Ethical considerations

Permission was obtained to conduct this study by the Government of Québec ethics committee (*Commission d’accès à l’information*), CORR internal review committee, and Maisonneuve-Rosemont Hospital ethics committee. Informed consent was waived.

## Results

**Figure 1:**
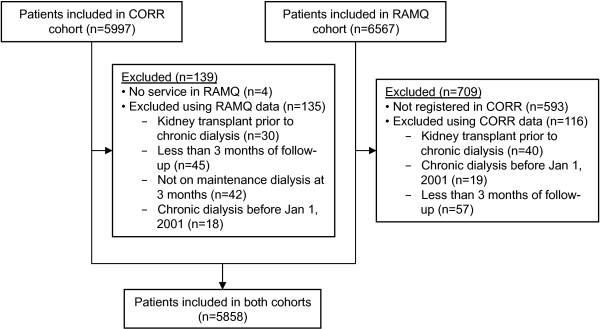
**Derivation of dialysis cohorts. **Despite meeting all inclusion criteria using RAMQ data, some RAMQ cohort patients were excluded because they were not registered in CORR (n=593) or had been excluded using CORR data (n=116). Similarly, some patients were excluded from CORR because they had no service in RAMQ (n=4) or were excluded based on RAMQ data (n=135).

As shown in Figure 1, 5997 patients were identified in the CORR cohort and 6567 patients in the RAMQ cohort. After merging both cohorts, 5858 patients remained in the study cohort. Median age was 68 years (interquartile range: 56 – 75), 39.7% were female, and the majority was on hemodialysis (84.2%) *versus* peritoneal dialysis (15.8%) at three months of dialysis initiation. Median follow-up time was 2.0 years (1.0 – 3.4) using ISQ data.

Among study patients, 1907 deaths were identified using CORR data, while 2074 were identified using ISQ data. In total, 96.4% of patients had a concordant death status: 3778 were still alive at the end of the study according to both data sources and 1872 had a concordant date of death (same or contiguous month). The remaining patients with non-concordant death status were distributed as follow: 6 were still alive in ISQ but deceased in CORR, 29 had a non-concordant date of death (by a median absolute difference of 120 days), and 173 patients were still alive in CORR but deceased in ISQ. Kappa statistics for concordance between dates of death was excellent: 0.92 (95% CI: 0.91, 0.93).

### All-cause and CVD mortality rates

**Figure 2:**
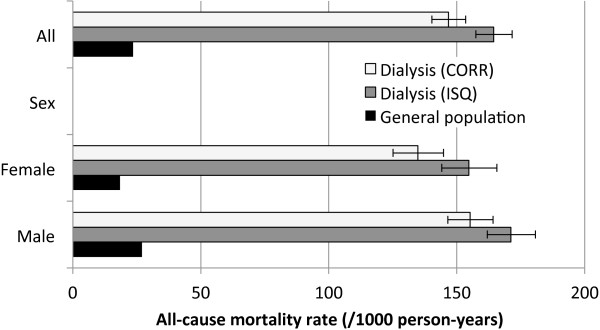
**All-cause mortality rates for patients receiving dialysis (using CORR *versus *ISQ data) and general population. **Rates for the general population are age- and sex-adjusted to the study population. Error bars represent 95% confidence interval of the rates. Abbreviations: HD, hemodialysis; PD, peritoneal dialysis.

**Figure 3:**
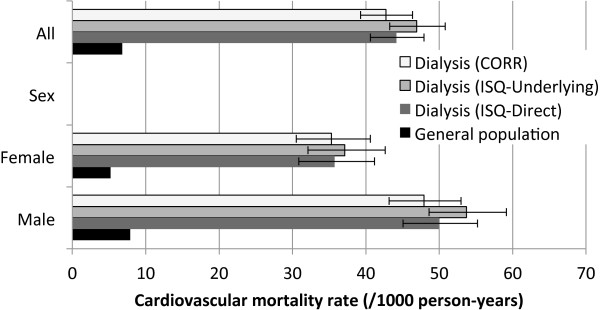
**Cardiovascular-related mortality rates for patients receiving dialysis (using CORR *versus *ISQ data) and general population. **Rates for the general population are age- and sex-adjusted to the study population. Error bars represent 95% confidence interval of the rates.

**Figure 4:**
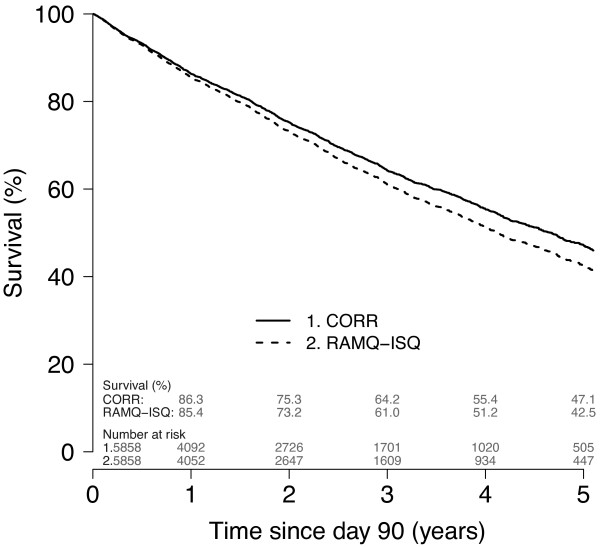
**Unadjusted Kaplan-Meier survival curve for all dialysis patients using CORR or ISQ data. **Note: Time starts at 90 day after dialysis initiation and the Kaplan-Meier plot was truncated when the number of patients at risk reached 10% of baseline.

All-cause mortality and CVD rates for the dialysis cohort (using CORR or ISQ dates of death) and GP are presented in Figures 2 and 3. Mortality rates were considerably higher in the dialysis population than in the GP. Mortality rate using CORR was lower when using ISQ (10.7%). Differences between CORR and ISQ dates of death also influenced the cumulative survival function, as shown in the Kaplan-Meier curve (Figure 4). CVD mortality rates were lower when using CORR data than using ISQ data, but the gap appeared smaller than with all-cause mortality.

### Causes of death distribution and concordance

Cause of death was missing or unknown in 25.6% of patients with a reported date of death in CORR, while this proportion was only 1.8% in ISQ. Distributions of causes for patients receiving dialysis and GP are presented in Table 2. Proportions of death attributed to either CVD or infections were higher using registry data than using underlying cause from death certificates. However, proportions of deaths from infections were similar between registry data and direct cause of death from death certificates. In comparison to the GP, use of death certificate data (underlying cause) resulted in a more conservative discrepancy in CVD deaths between dialysis and GP groups: *e.g.* in men, the difference in percentage points was reduced from 12% (41.5%-29.3%) to 3% (32.1%-29.3%), and similarly in women it was reduced from 7% (35.2%-28.2%) to -4% (24.3%-28.2%). Differences in percentage points for infections were also reduced to 1.7% (5.0%-3.3%) for men and 1.2% (4.8%-3.6%) for women when using death certificate data (underlying cause).

**Table 2:** Distribution of causes of death according to CORR and ISQ for patients receiving dialysis and for the general population

**Causes of death**	**CORR**		**ISQ – Underlying cause**		**ISQ – Direct cause**		General population*
	**N**	**%**	**N**	**%**	**N**	**%**	**%**
*Both sex*							
Cardiovascular	555	39.1%	592	29.1%	557	27.4%	28.9%
Infection	149	10.5%	101	5.0%	222	10.9%	3.4%
Malignancy	149	10.5%	284	14.0%	153	7.5%	36.1%
Other	565	39.9%	1059	52.0%	1104	54.2%	31.6%
Diabetes	N/A		339	16.7%	14	0.7%	3.3%
Kidney failure	N/A		400	19.6%	576	28.3%	1.8%
*Female*							
Cardiovascular	189	35.2%	191	24.3%	184	23.4%	28.2%
Infection	62	11.6%	38	4.8%	92	11.7%	3.6%
Malignancy	59	11.0%	107	13.6%	59	7.5%	34.9%
Other	227	42.3%	449	57.2%	450	57.3%	33.3%
Diabetes	N/A		146	18.6%	7	0.9%	3.4%
Kidney failure	N/A		161	20.5%	232	29.6%	1.6%
*Male*							
Cardiovascular	366	41.5%	401	32.1%	373	29.8%	29.3%
Infection	87	9.9%	63	5.0%	130	10.4%	3.3%
Malignancy	90	10.2%	177	14.2%	94	7.5%	36.7%
Other	338	38.4%	610	48.8%	654	52.3%	30.7%
Diabetes	N/A		193	15.4%	7	0.6%	3.2%
Kidney failure	N/A		239	19.1%	344	27.5%	1.8%

Note: Cause of death was missing or unknown in 489 patients (25.6%) in CORR and 38 patients (1.8%) in ISQ. Kidney failure and diabetes are not defined in CORR internal coding scheme and could not be reported.

*General population of Quebec, Canada, 2001 to 2007, indirectly standardized.

Using the underlying cause of death, 51.4% of patients who had a cause of death reported in both CORR and ISQ had congruent death categories (Table 3). Kappa statistic was fair at 0.27 (95% CI: 0.23, 0.31). The chi-square test comparing proportions of concordant causes of death between categories was highly significant (p<0.001), suggesting that the concordance in causes of death between CORR and ISQ data did not occur by chance. Concordance for causes of death between CORR and ISQ improved when the direct cause of death was used (Table 4). Percent agreement increased to 57.9% and kappa statistic remained fair at 0.36 (0.33, 0.40).

**Table 3:** Causes of death for patients with a cause of death in both CORR and ISQ using the underlying cause of death in ISQ

		**ISQ -- Underlying cause**				
		**Cardiovascular**	**Infection**	**Malignancy**	**Other**	
CORR	Cardiovascular	237	19	13	274	543 (39.1%)
	Infection	24	35	15	74	148 (10.7%)
	Malignancy	16	1	106	25	148 (10.7%)
	Other	129	17	67	336	549 (39.6%)
		406 (29.3%)	72 (5.2%)	201 (14.5%)	709 (51.1%)	1388 (100%)

**Table 4:** Causes of death for patients with a cause of death in both CORR and ISQ using the direct cause of death in ISQ

		**ISQ -- Direct cause**				
		**Cardiovascular**	**Infection**	**Malignancy**	**Other**	
CORR	Cardiovascular	286	32	5	220	543 (39.1%)
	Infection	14	69	3	62	148 (10.7%)
	Malignancy	10	12	68	58	148 (10.7%)
	Other	85	44	39	381	549 (39.6%)
		395 (28.5%)	157 (11.3%)	115 (8.3%)	721 (52.0%)	1388 (100%)

Among the 173 patients who were considered still alive in CORR but deceased in ISQ, 17.3% of deaths were attributed to CVD (underlying cause in ISQ database), 2.3% to infection, 22.0% to malignancy, 56.6% to other causes, and 1.7% were missing.

### Sensitivity analyses

Removing codes possibly overlapping multiple categories (see *Methods*) did not improve concordance (kappa: 0.31 with underlying and 0.35 with direct cause). Reclassifying missing cause of death in CORR as CVD (kappa: 0.20 with underlying and 0.26 with direct cause) or other causes (kappa: 0.22 with underlying and 0.30 with direct cause) led to slightly worse results.

## Discussion

This study reports on the comparison of cause of death proportions between dialysis patients and the GP. It demonstrated poor concordance between registry data and death certificates. This discrepancy between registry data and death certificates led to different conclusions when proportions of death attributed to CVD were compared between the dialysis population and the GP. While CORR showed a higher CVD death proportion among dialysis patients, ISQ showed almost no difference compared to the GP. While the proportion of deaths due to infection was also higher among dialysis patients using CORR data than when compared to underlying cause of death, proportions estimated from direct cause of death in the death certificate showed that they remained an important complication of disease (biological mechanism) leading to death. Finally, cause of death was missing in more than a quarter of deaths in CORR, greatly diminishing its usefulness.

Many countries and regions collect and report mortality data from renal dialysis programs using registries. Examples include the United States Renal Data System (USRDS), United Kingdom Renal Registry (UKRR), European Dialysis and Transplant Association/European Renal Association (EDTA/ERA), and Australia and New Zealand Dialysis and Transplant Registry (ANZDATA) [18]. Most dialysis registries use an internal coding system to classify cause of death instead of ICD-10 [2,19]. Reports of distribution of causes of death for patients on dialysis vary between countries, influenced in part by different age distributions and case-mix among populations. CVD remains the most frequent cause in all (31 to 55% of deaths), followed in most countries by infections (11 to 18%), and malignancies (4 to 13%) [19-21]. Of note, distribution of causes of death reported for the GP are age and sex adjusted to the dialysis cohort. Therefore, causes of death associated with older age have a higher weight, explaining why malignancies are the leading cause of death at 36.1% in the GP.

Our finding of only fair concordance of cause of death between ISQ and CORR is consistent with results from previous reports using USRDS and ANZDATA (overall kappa from 0.22 to 0.24) [6,7]. One may hypothesize that, in most of those dialysis populations, reported CVD death proportions are likely higher than what would have been reported using DC. To our knowledge, no study has quantitatively evaluated the proportion of deaths attributable to different causes in comparison to GP, using the same source of information (DC) for both populations.

In the UKRR, part of their data (England and Wales) is linked with national vital statistics [18,22]. In USRDS, only 1% of deaths are ascertained through national vital statistics [2]. However, reporting death to USRDS is mandatory. CORR is a voluntary registry with no linkage to vital statistic registries. This may account for some of the discrepancies observed in our analysis. Given that information from registries (such as cause of death) is precious to guide policy, planning and resources we insist that better identification of true causes of death in dialysis populations is important, and that we do have an opportunity to remedy to the current situation in Canada.

While validity of death occurrence and its date is fairly high in vital statistic registries such as ISQ [23], misclassification of the cause of death is known to occur [7,24,25]. Of note, neither CORR nor ISQ is a true gold standard for cause of death, which will always be subject to some misclassification. DC, obtained from the ISQ data, constitute the most valid practical tool available to determine the cause of death on a provincial level. This study did not evaluate differences between registry and death certificates coding variation by country. However, as mentioned earlier poor concordance was reported in two other countries, so it is likely that this is a true phenomenon.

## Conclusion

In conclusion, evaluation of causes of death using DC in dialysis patients in Quebec revealed that they do not have substantially different proportion of death due to CVD or infections than the GP. However, infections appeared to be a frequent complication or biological mechanism leading to death. While all-cause and CVD absolute mortality rates remain many times higher among dialysis patients compared to the GP (no matter which source of data), use of registry data alone would lead health care practitioners and researchers to conclude that there is more than 10 percentage points higher proportion of deaths attributed to CVD in dialysis populations than is really the case. In an era of increasing need for accuracy in medical records, we believe that validation of registry data, or development of better linkage with vital statistics in Canada, should be undertaken for dialysis populations. In this way, we may be able to better establish priorities for prevention and therapeutic efforts, or clinical and research resources allocation. While CVD remains an important issue among dialysis patients, infections appear to be an important target to consider as a means to reducing mortality in that population.

## Competing interests

This work was supported by a Fonds de la recherche en santé du Québec (FRSQ) operating grant. Dr. Lafrance is supported by a KRESCENT New Investigator Award (public funds). The authors have no other competing interests.

## Authors’ contributions

JP L (conception and design, analysis and interpretation of data, and writing of manuscript), ER (analysis and interpretation of data, and critical appraisal of article), SI (interpretation of data and critical appraisal of article), ML (interpretation of data and critical appraisal of article), VP (interpretation of data and critical appraisal of article), NE (analysis and interpretation of data and critical appraisal of article), and MV (interpretation of data and critical appraisal of article). All authors read and approved the final manuscript.

## Pre-publication history

The pre-publication history for this paper can be accessed here:

http://www.biomedcentral.com/1471-2288/13/51/prepub
